# Determine the quality of human embryonic stem colonies with laser light scattering patterns

**DOI:** 10.1186/1480-9222-15-2

**Published:** 2013-01-14

**Authors:** Chi-Shuo Chen, Matthew Biasca, Catherine Le, Eric Y-T Chen, E Daniel Hirleman, Wei-Chun Chin

**Affiliations:** 1 Bioengineering, School of Engineering, University of California, Merced, CA, USA; 2 School of Engineering, University of California, Merced, CA, USA; 3 School of Natural Sciences, University of California, Merced, CA, USA

**Keywords:** Light-scattering, Human embryonic stem cell, Pluripotency, Label-free detection

## Abstract

**Background:**

With the prompt developments of regenerative medicine, the potential clinical applications of human embryonic stem cells have attracted intense attention. However, the labor-intensive and complex manual cell selection processes required during embryonic stem cell culturing have seriously limited large-scale production and broad applications. Thus, availability of a label-free, non-invasive platform to replace the current cumbersome manual selection has become a critical need.

**Results:**

A non-invasive, label-free, and time-efficient optical platform for determining the quality of human embryonic stem cell colonies was developed by analyzing the scattering signals from those stem cell colonies. Additionally, confocal microscopy revealed that the cell colony morphology and surface structures were correlated with the resulting characteristic light scattering patterns. Standard immunostaining assay (Oct-4) was also utilized to validate the quality-determination from this light scattering protocol. The platform developed here can therefore provide identification accuracy of up to 87% for colony determination.

**Conclusions:**

Our study here demonstrated that light scattering patterns can serve as a feasible alternative approach to replace conventional manual selection for human embryonic stem cell cultures.

## Background

There have been increasing interests in the applications of human embryonic stem cells (hESCs). These hESCs present almost unlimited applications and opportunities for future advances in biotechnology and regenerative medicine. Through the research of regenerative medicine, specific functional cell types have been differentiated from hESCs for stem cell-based therapies [1,2]. For instance, advanced protocols have been developed to enrich the functional cardiomyocytes differentiated from hESCs for the treatment of irreversible cardiac tissue damage [3]. In addition, ESCs-derived cardiomyocytes were implanted into adult dystrophic mice which formed intracardiac grafts after 7-weeks [4]. Stem cells can furthermore be used to provide functional neuronal cells for potential treatments of neurodegenerative disorders such as Alzheimer’s disease, Huntington’s’ disease, and spinal cord injury [1,5]. Specifically, dopaminergic neurons differentiated from ESCs have shown to offer partial Parkinson’s disease recovery in animal models [6,7]. Moreover, functional islet-like cells derived from ESCs have been demonstrated to respond to glucose and produce insulin, which hold promising potential for treatments of diabetes [8]. In addition to regenerating tissue replacements, human stem cells and/or differentiated cells can serve as promising platforms for drug discovery and toxicity testing within the pharmaceutical industry. These human cell-based platforms not only provide human pathology models, but are also important platforms for evaluating new drug compounds in human physiological environments. For example, differentiated hepatocytes have been used for drug metabolism studies in preclinical drug discovery; additionally, cardiomyocytes differentiated from hESCs have been utilized for cardiac drug discovery and cardiac safety assessments [9]. Likewise, undifferentiated hESCs can provide a model for embryotoxicity testing [10].

To fully develop these promising industrial applications, one of the most crucial issue is the maintenance and expansion of the self-renewing undifferentiated hESCs that are able to retain the capacity to differentiate into desired cell types [10]. In order to maintain the pluripotency of hESCs in cultures, manual microdissection is broadly used for cell passaging, which requires laborious protocols and quality-controlled manual selection [9]. Typically, according to the protocols established by National Stem Cell Bank (NSCB) and biotechnology companies [11,12], undifferentiated hESC cell colonies need to be manually selected and transferred to new plates every 7 ~ 10 days. The quality of undifferentiated hESC colonies is determined with bright-field/phase contrast light microscopy based on human-experience/assessment. Various rating scales/criteria have been established mainly based on the morphology of individual colonies [13]. Chiefly, good quality (good) hESCs, without stacking nor undesired differentiations, grow as uniform flat colonies with clear colony edges; low quality (bad) hESC colonies show various shapes with visible surface structures and irregular edges. The quality of hESCs plays a critical role in the downstream applications. Consequently, determining the undifferentiated hESC cell colonies by this manual selection process serves as an essential step in subculture; only high-quality undifferentiated colonies should be transferred; conversely, transferring poor-quality colonies results in the lost of pluripotency [13]. Nonetheless, this time-consuming and labor–intensive manual selection can hinder the development of large-scale culture for practical/clinical applications. Moreover, lack of standard criteria for rapid determination of stem cell qualities may lead to the quality control/assurance issues of industrial-scale reproducibility for therapeutic applications [14].

A critical requirement for hESC research and technological developments is the efficient assessment of cell differentiation status. To address this issue, several approaches have been developed [13,14]. For instance, laser flow cytometers have been broadly used to provide comprehensive information of stem cell differentiation with the use of fluorescent cell surface antigens or protein markers such as Oct-4 and Nanog, or SSEA-3 in hESCs [13]. To further simplify specimen preparation and lower the immunohistochemistry costs, the microfluidic dielectrophoresis (DEP) platform have been introduced. Based on different cellular dielectric properties, without cell-type specific markers, neurons and astrocytes differentiated from mouse neural precursor cells can be detected and isolated in microfluidic channels (500 μm in width and 50 μm in height) [15]. However, these current technologies may not be appropriate for hESC colony quality evaluation, since hESC colonies would be squeezed within sheath flow and broken into undesired small pieces while they are transported in the cytometry pipe line or microfluidic channels. As a result, the dispersion of cell colonies may further complicate the subculture process.

To maintain the colonies’ integrity during the selection process, advanced image analysis systems have been developed to analyze the cell colonies (STEMvision, STEMCELL technologies, Vancouver, Canada). With methylcellulose-labeled assay, images of hematopoietic colonies can be acquired and scored automatically. However, excessive labeling assays and the requirement of specific cultureware may hinder the large-scale hESC technological developments and have an unknown influence on hESC differentiation capacities.

**Figure 1:**
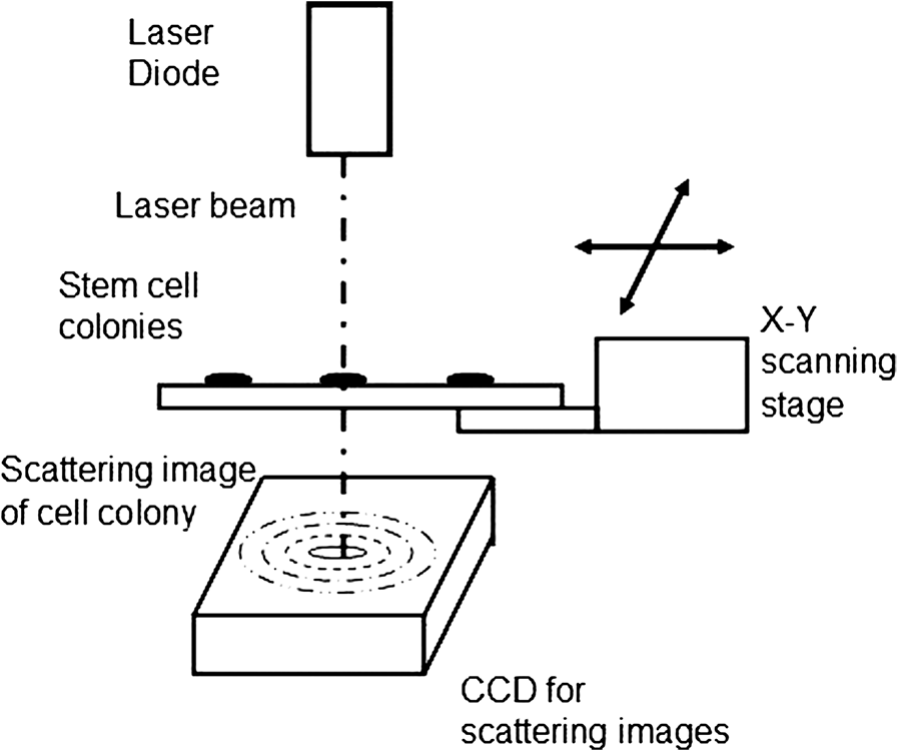
**Sketch of optical system.** In this optical system, laser light (wavelength = 633 nm) was guided to incident on the hESCs colony perpendicularly. Forward scattering pattern of the colony was collected with CMOS sensor in the back of culture dish. During the scanning step, specimens were tracked and transported automatically by motor stage.

In order to create a label-free, non-invasive platform to evaluate the quality of stem cell colonies, we introduced optical forward-scattering technology in this study (Figure 1). Previously, scattering technology has been applied to assess micron spheres on silicon wafers [16]; it can also be applied to identify collected microbial contaminants from different food sources [17]. Various characteristics of bacterial colonies, such as colony matrices or shapes of colonies, govern the interaction between incident light and the colonies resulting in different scattering patterns (signatures) [18]. However, the feasibility of rapid cell selection with scattering patterns has not yet been developed for human stem cells. In this study, one of the most used stem cell lines, H9 (WiCell), was selected as the model line [19]. We demonstrated the feasibility of optical forward-scattering in determining the quality of hESC cell colonies. Furthermore, standard immunohistochemistry assay (Oct-4) was performed on hESC colonies to confirm the results from the newly developed light scattering identification protocol.

## Results and discussion

**Figure 2:**
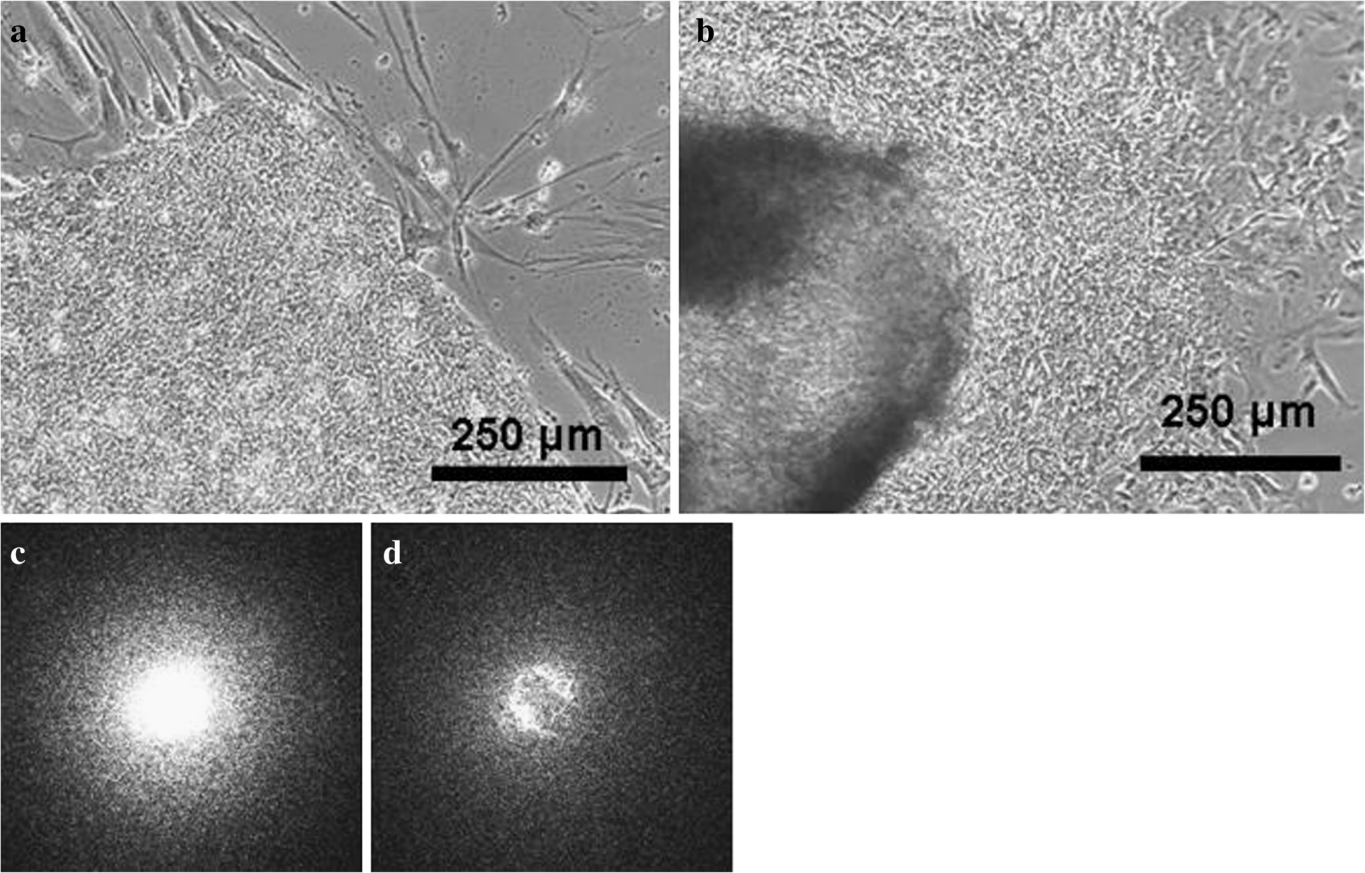
**Forward-scattering patterns of hESCs colonies.** A single colony was illuminated with a laser beam and the scattering patterns were projected on the detectors. The light intensity of scattering patterns created by good colonies (**a**) showed more homogeneous distribution (**c**) compared to those from bad colonies (**b**, scattering patterns in **d**).

In order to establish the computer-based recognition database to identify good/bad colonies, hESC colonies were first manually categorized into good/bad sets. Representative colonies images were presented with phase contrast microscopy (Figure 2a and b). Scattering patterns of 290 pre-categorized colonies were collected into the database to establish good/bad colony criteria. Representative forward scattering patterns from good/bad colonies are displayed in Figure 2c and d. Compared to the scattering patterns of bad hESC colonies, the light scattering pattern of good hESC colonies displayed a more symmetrical intensity distribution. In contrast, irregular or patchy scattering patterns were observed in most of the bad hESC colonies.

Conventionally, one of the important criterion for evaluating the quality of hESCs has been the determination of colony morphology by manual evaluation with light microscopy. Since colony morphology will likewise influence the scattering patterns of hESC colonies, we aim to utilize this connection to determine colony quality from scattering patterns. In order to evaluate these key attributes, we used laser scanning confocal microscopy to investigate the morphology of hESCs colonies with different qualities. Optical-sliced images of DAPI stained cells were collected and reconstructed to reveal detailed 3-dimensional (3-D) hESC colonies. Results of 3-D z-stack measurements showed the average height was 53 ± 4.2 μm of good colonies and was 44 ± 13.9 μm of bad colonies. Student *t*-test was applied to compare the different heights and indicated the significant height difference (p-value < 0.01) between good/bad colonies. The larger variations of height measurements also indicate the non-uniform stacking microstructures within bad colonies, rather than the homogenous spatial distribution of cells within good colonies. Our 3-D colony image analysis provided consistent quantified assessments that were supported by the outcomes from the human experience-based evaluation.

As in the diffraction patterns created when light propagates through optical apertures, the amplitudes and phases of light are modulated when light passes through the biological specimens [18]. In previous studies [17,20], it has been shown that the central thickness and radius of bacterial colonies may dominate the scattering pattern formations. Served as the superposition of various apertures, the observed non-even spatial colony variations can lead to the non-uniform scattering patterns.

In order to establish the colony characteristics database, advanced images analyses were performed to further analyze scattering patterns. Each scattering image features were extracted utilizing 2-D Zernike moment invariants [21,22]. In general, the center of the image was set as the origin and pixels were mapped into a specific coordinate, then the image boundary and characteristic contents were quantified with a set of complex Zernike moment polynomials [22]. The 161 feature vectors were selected with Fisher’s criterion to represent the image characteristics in order to optimize classification outcomes [17]. The classification was performed with Support Vector Machine (SVM)-based algorithm; a pattern recognition method has been widely used in handwritten recognition, objective recognition, and face detection [17,23,24]. In order to investigate the precision of our computerized identification protocol for good/bad colony identification, additional 100 manually-classified colonies were assessed with the BARDOT (Bacterial Rapid Detection using Optical scattering Technology) system under an optimized system setting, which was the same setting for database training. Scattering patterns were analyzed with the same setting parameters for these testing samples. As shown in Table 1, 87% of manually-classified-good colonies could be determined into good-colony classification, and 83% of manually-classified-bad ones could be resolved. Factors causing false determination may come from either the manual selection process or the current system set-up. As discussed, manual selection is based on researcher’s experience and this individual human bias may lead to certain false determination. Larger image variation caused by the heterogeneous colony shapes, can also lead to the false classification outcome. In spite of this, an improved image analysis algorithm may help to decrease the false determination rate as well.

**Table 1:** Distinguish rate (%) of good/bad colonies

**BARDOT determined**	**Good hESCs colonies**	**Bad hESCs colonies**
**Human categorized**		
**Good hESCs colonies**	**87.3%**	**12.7%**
**Bad hESCs colonies**	**16.4%**	**83.6%**

**Figure 3:**
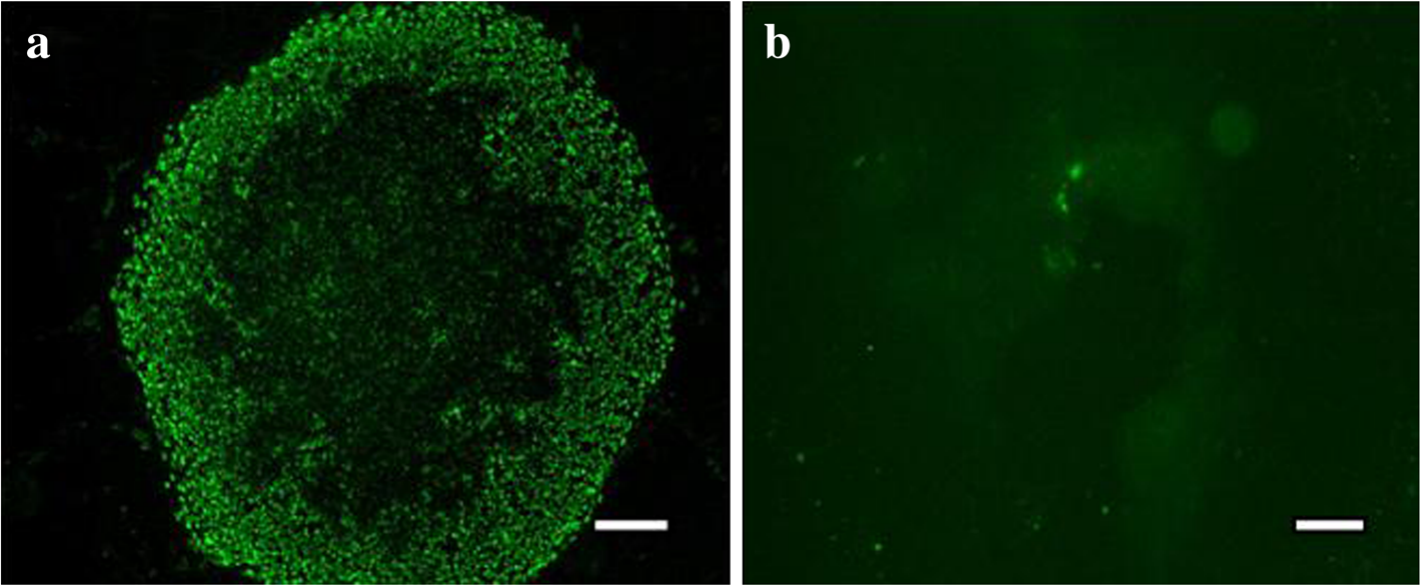
**Fluorescent images of hESCs colonies.** Immunohistological staining assay was applied to determine the qualities of hESCs colonies. Anti-Oct-4 antibody was used to label the self-renewing pluripotent stem cells in colonies. Results indicated the existence of more Oct-4 marked cells in categorized good hESCs colonies (**a**) versus those of bad hESCs colonies (**b**).

**Figure 4:**
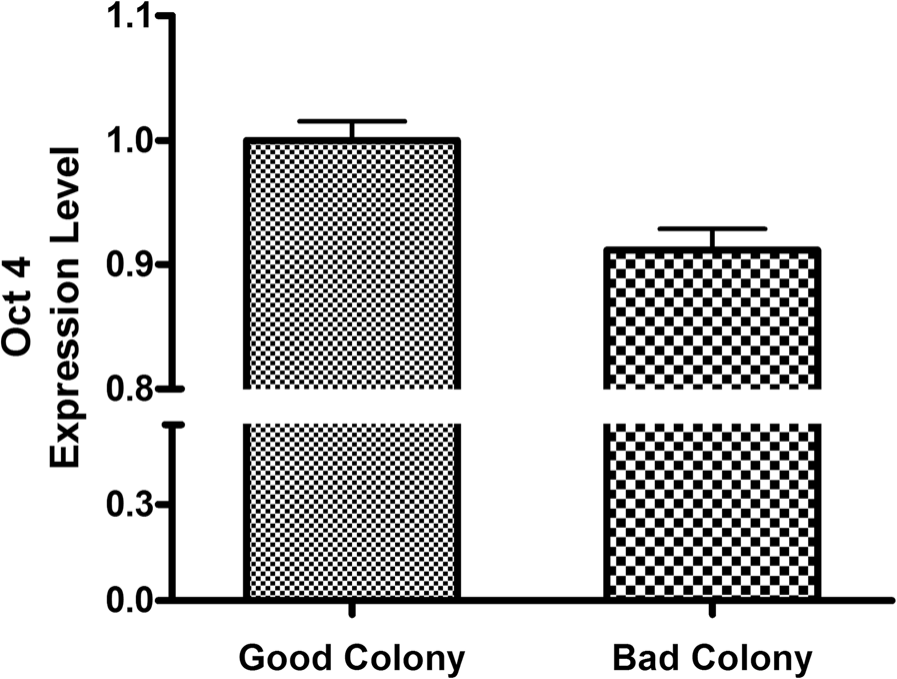
**Expression levels of Oct-4/DAPI in hESCs colonies.** Colonies categorized into good/bad colonies showed different Oct-4/DAPI expression ratios. The expression was statistically different in good/bad classifications (p-value < 0.001). Higher Oct-4/DAPI expression ratios indicated a higher percentage of pluripotent stem cells in the classified-good colonies.

In addition to testing the accuracy of BARDOT determination with manually classified specimens, standard immunological staining was also used to quantify the classification outcomes. Oct-4 is an important transcription factor during cellular development that serves to regulate the pluripotency and self-renewal of embryonic stem cells. The decrease of Oct-4 expression levels has been widely used as an indicator for hESC differentiation (loss of pluripotency) [25,26]. To assess pluripotency of the classified stem cell colonies, colonies were labeled with anti-Oct-4 (stem cell pluripotency biomarker) and then imaged with epi-fluorescent microscopy (N = 18, from each classification). The Oct-4 expressions were subsequently assessed with image analysis software (ImageJ, NIH). The fluorescence of DAPI staining (DNA stain) was used to quantify the cell number within a single colony. Representative fluorescent images of Oct-4 and DAPI are shown in Figure 3. Here, we used the Oct-4 to DAPI expression ratio to indicate the pluripotency of cell colonies (Figure 4). Our results showed a higher Oct-4/DAPI expression ratio which was observed in classified-good colonies, rather than classified-bad ones (Oct-4/DAPI ratios were significantly different, p-value < 0.001). Noticeably, due to the heterogeneous cell populations within the colony, certain portions of hESCs within classified-bad colonies expressed Oct-4 that can reduce the difference of Oct-4/DAPI expression level between good/bad classes. These small expression differences not only demonstrate the complexity in evaluating the heterogeneous colonies quality with immunostaining, but also highlight the unique advantage of light scattering assay having high sensiti-vity. Moreover, it has been shown that extracellular matrix properties can alter, resulting in a change to a cell’s organization during the progression of cell differentiation [27,28]; accordingly, the refractive index and the microstructure of colonies can contribute to the variations of scattering patterns [17,29]. The accuracy of this scattering system could be further improved by taking these parameters into consideration.

## Conclusions

In this study, we demonstrate the feasibility in determining the quality of hESC cell colonies with optical forward-scattering technology. Integrating with SVM machine learning, this technology provides a critical module for automatic hESC cell colony selection. Without any biochemistry labeling processes or manual labor, the determination results from our protocol showed high correlation with the results of standard immunohistochemistry assay. In addition, this new label-free light scattering process significantly reduces the cost and time for specimen preparation. Though only two fundamental categories of the hESC database were assessed here, our results demonstrated that the light scattering patterns could provide unique signature standards for hESC categorization. Additionally, this non-invasive optical protocol demonstrates its potential capacity for applications on other various stem cell colony types with specific differentiation lineages, such as iPS (induced pluripotent stem) cells, neuronal stem cells, cells grown on Matrigel, etc. Due to the unique simplicity of this non-destructive technology, the hESC cell colonies scoring protocol developed in this study is expected to provide a general calibrator to facilitate industrial scale productivity of consistent quality stem cells for applications of regenerative medicine.

## Methods

### Human stem cell culture

(Madison, WI, passage 32–60) were cultured following the NSCB protocols. Briefly, undifferentiated hESC were maintained on feeder cell layers, which were mitomycin C treated mouse embryonic fibroblasts (MEF). Serum-free medium was used in this experiment and the medium was composed of DMEM-F-12, Knockout Serum Replacement, basic fibroblast growth factor, L-glutamine, and MEM non-essential amino acid [30]. Medium was changed daily and hESCs were manually selected to pass every 7 days. All hESC colonies were analyzed on day 7 to acquire forward light scattering patterns in order to eliminate the variations from culture conditions.

### Laser forward-scattering system and image analysis

In this study, an automated BARDOT system was modified for hESC colony scanning. This system was composed of a laser diode (0.95 mW, 632 nm), monochromatic CMOS image sensor, and an x-y scanning stage. The laser beam was illuminated on a single colony with computer-aided positioning. The resulting forward-scattering signals were collected with a CMOS image sensor. To analyze the features from colony scattering patterns, Pseudo-Zernike moments (PZMs) were applied in our current models. Support vector machine (SVM)-based algorithm was employed for recognition and classification of the images [17,29]. In order to establish the colony characteristic database, 290 colonies were scanned with BARDOT. Their scattering patterns were then collected and analyzed.

### Immunohistological staining

In order to quantify pluripotency, after culturing in medium for 7 days, hESCs were fixed with 4% wt paraformaldehyde for 20 min at room temperature. Paraformaldehyde was removed with 3 subsequent PBS rinses. Triton-X (0.1%, Sigma) was used to penetrate cell membranes. The sample was then incubated in 2% BSA for 40 min to block non-specific binding. Primary antibody anti-Oct-4 (Millipore, 1:100) was used to identify pluripotent stem cells within colonies [13]. Alexafluor-488 conjugated antibody was used for secondary antibody staining. All samples were stained with 4^′^, 6-diamdino-2-phenylindole (DAPI, Invitrogen, 1:3000) for quantifying cell numbers.

### Fluorescent imaging of hESC colonies

Colony images for Oct-4 quantities analysis were collected with fluorescent microscopy. Fluorescence of Alexafluor-488 was excited at 488 nm and the emission was collected between 500 nm and 560 nm. DAPI staining signals were collected to identify the cell numbers. In this study, images of colonies were categorized into two separated groups by BARDOT collection (N = 18 in each classification). Laser scanning confocal microscopy was utilized to investigate the surface morphology and colony homogeneity of good/bad colonies.

## Abbreviations

BARDOT: Bacterial Rapid Detection using Optical scattering Technology; BSA: Bovine serum albumin; DAPI: 4′, 6-diamdino-2-phenylindole; hESCs: Human embryonic stem cells; MEF: Mouse embryonic fibroblasts; PBS: Phosphate buffered saline; PZMs: Pseudo-Zernike moments; SVM: Support vector machine.

## Competing interest

Drs. Chen, Chin and Hirleman are listed as co-inventors of a patent application filed by UC Merced based on this study.

## Authors’ contributions

CSC, WCC, EYTC and EDH conceived and coordinated the study and drafted the manuscript. MB and CL performed some of the stem cell culture and light scattering experiments. CSC performed most of the research and analyzed the data. All authors read and approved the final version of this manuscript.
